# COVID-19 Double Annual Epidemic Peaks in Summer and in Winter from 2022, Irrespective of the Rate of Mask Wearing and Vaccination

**DOI:** 10.3390/v17121612

**Published:** 2025-12-13

**Authors:** Shinako Inaida, Richard E. Paul, Minsoo Kim

**Affiliations:** 1 Laboratory of Integrative Molecular Medicine, Graduate School of Medicine, Kyoto University, Kyoto 606-8303, Japan; 2 Department of Preventive Medicine and Behavioral Science, Kindai University Faculty of Medicine, Osaka 590-0197, Japan; 3 Pasteur Kyoto International Joint Research Unit for Integrative Vaccinomics, Kyoto 606-8303, Japan

**Keywords:** COVID-19, epidemic, incidence rate, vaccine

## Abstract

Although vaccination for COVID-19 and mask wearing were two of the main preventive measures against infection, their impact is unclear. In the present study, by using national surveillance data in Japan, we compared the incidence rate and weekly case increase ratios of COVID-19 with the domestic stocks of masks and vaccination coverage. The trajectory of epidemic growth increased rapidly in the summer of 2021, concomitant with the launch of the mass national vaccination program. The most rapid spread of the epidemic was found in 2022, approximately 6 months after the national mass vaccination started, with the emergence of the Omicron variant. From 2022, two annual epidemic peaks occurred with seasonal changes. Whilst the winter peak follows the expected seasonal trend in respiratory infections, the summer peak may reflect a combination of short-term herd immunity and behavioral patterns. Nevertheless, these epidemic peaks continued irrespective of vaccine coverage and mask use. Further analysis into the duration of protective efficacy of the vaccines and mask use is required.

## 1. Introduction

The recent COVID-19 pandemic has underlined the importance of being prepared for the next pandemic. However, questions remain concerning the efficacy of the implemented prevention methods, notably mask wearing and vaccination [1]. In Japan, the dominant variant of the SARS-CoV-2 virus was the Delta variant in August 2020, and then the Omicron variant from 2022 [2]. Omicron has since been shown to be an immune escape variant with evasion of neutralization using sera from vaccinated individuals [2,3]. The extent to which this impacted the epidemic rate needs to be clarified [4]. The effect of mask wearing has been the subject of several studies with respect to infection risk and reproduction number [1]. However, because hand washing and other protective methods were implemented simultaneously, examining the effect of mask wearing alone is difficult. Meanwhile, mask wearing has been suggested to be associated with a bioaccumulation of microplastics in the human body; microplastics of disposable medical face masks, including polypropylene (PP), polyethylene (PE), polycarbonate, polyester/polyethylene terephthalate, polyamide/Nylon, polyvinylchloride, and ethylene-propylene copolymer have been detected in organs [5,6]. Another study found increased levels of PE, PP, and polyvinyl chloride (PVC) in the brain, kidney, and liver in decedent tissue samples [7]. The amount of microplastics in such tested tissue samples increased more rapidly during the COVID-19 pandemic years, with a 50% significant increase in 2024 samples as compared with 2016 samples of brain tissues [7]. This risk puts more emphasis on establishing the benefits of mask wearing to prevent disease spread. In light of the uncertain efficacy of the vaccine against the Omicron variant and the unknown impact of mask wearing, we compared the epidemic pattern with vaccination coverage and mask use.

## 2. Methods

We used the national surveillance data in Japan for COVID-19 cases between January 2020 and March 2025 [8,9]. In Japan, all cases of COVID-19 were collated until May 2022, and then the surveillance was switched to sentinel surveillance (as for influenza) [10]. Sentinel surveillance (for influenza) is conducted in ~5000 clinics across the country, which have been operating for more than 40 years [8]. We estimated the number of COVID-19 cases using sentinel surveillance data by using the statistics of these clinics [10]. The incidence rate of infection (percentage of the population infected) was calculated for each epidemic wave and year. We calculated the weekly case increase ratio (the ratio between the number of cases of the current week and the number of cases of the previous week). This weekly epidemic growth was calculated from five weeks until one week before the epidemic peak (the week when the highest incidence rate was recorded), thereby capturing the growth phase [11]. We also compared the yearly mask stocks between 2019 and 2024 (by using data on the domestic stock of masks for citizens) [12]; the time between being stocked in the warehouse before selling is relatively short and therefore provides at least a proxy for mask use. The vaccination coverage was taken using the national statistics data. Vaccination for first and second doses began for medical and elderly care workers in April 2021 and was completed for them by the end of July. Vaccination of citizens started in June 2021 [13]. Data for vaccination doses were available online up to March 2024 [13,14,15]. Statistical analysis was conducted by using SPSS v29 (IBM Corporation, Chicago, IL, USA).

## 3. Results

**Figure 1:**
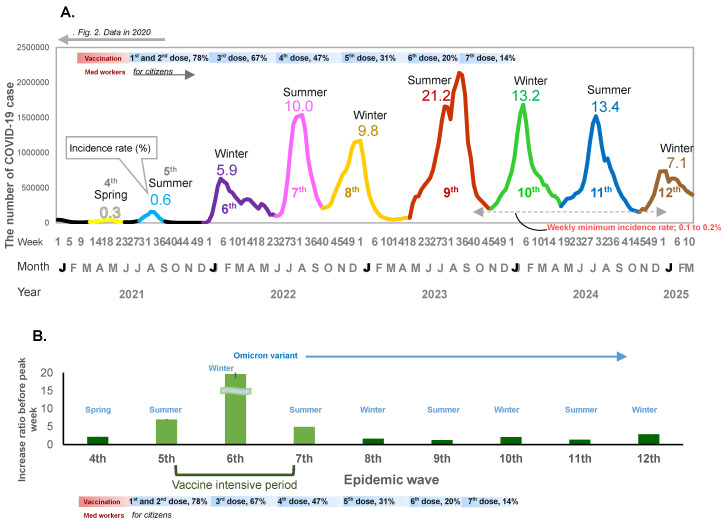
(**A**) The number of COVID-19 cases for the no-dose and first-to-seventh-dose vaccination periods. The weekly number of COVID-19 cases, incidence rate of infection, and vaccination periods are shown. The X-axis shows the epidemiological week, month, and year. The y-axis shows the weekly number of cases. The horizontal bar shows the vaccine phases. The line graph shows the fourth to the twelfth epidemic waves. Decimal numbers are the crude incidence rates (%) per population (0.12 billion). for each wave. (**B**) Weekly case increase ratio for 5 weeks before the peak week (means and 95% Confidence intervals).

**Figure 2:**
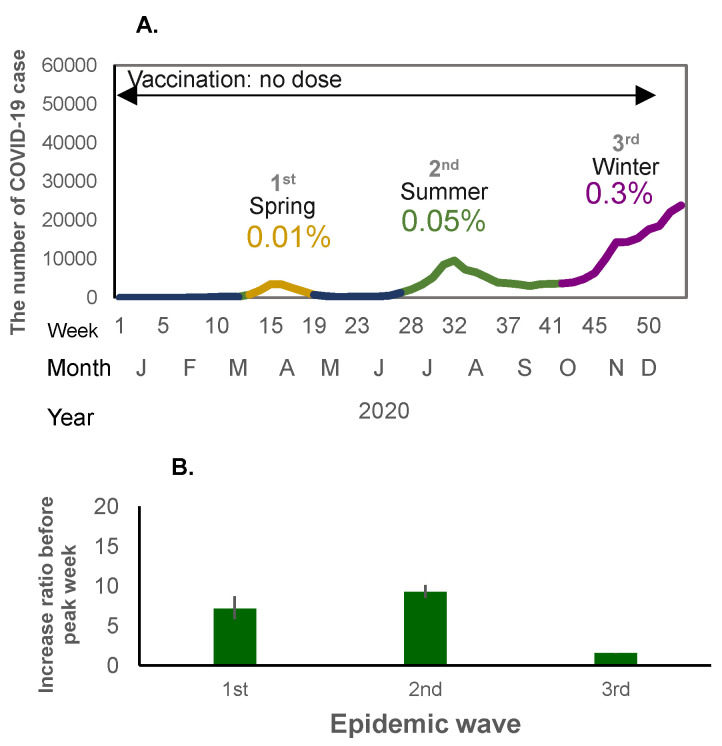
(**A**) The number of COVID-19 cases in the first to third waves of the pandemic (no-dose vaccine period) in 2020. The X-axis shows the epidemiological week, month, and year, and the Y-axis shows the number of COVID-19 cases. The percentage shows the crude incidence rates (%) per population (0.12 billion) for each wave. (**B**) Weekly case increase ratio for the corresponding period (means and 95% Confidence intervals).

When mass national vaccination started in June 2021, both the incidence rate of infection and the weekly case increase ratio started to increase (Figure 1A,B and Figure 2A,B). There was a relatively large epidemic increase in the beginning of 2022; the incidence rate of COVID-19 suddenly increased as compared to previous waves: 0.01% (first wave in 2020), 0.06% (second wave in 2020), 0.3% (third wave in 2020 and fourth wave in 2021), rising to 0.6% (fifth wave in 2021) when the mass vaccination started, and then further increasing to 5.9% (6th wave in 2022) during the following winter (Figure 1A and Figure 2A). This increase occurred immediately after completion of the second vaccine dose (79.0% for the first dose and 77.5% for the second dose) (Figure 1A). The weekly case increase ratio in the 5 weeks before the epidemic peak week increased 195 times in the sixth wave at the beginning of 2022, when the Omicron variant became predominant. The average of the weekly case increase ratio prior to vaccination implementation (third and fourth waves) was 1.85 (95% confidence interval [CI]: 1.53–2.17]), and the average of fifth and seventh waves covering the period during and after the implementation of intensive vaccination was 5.94 (95%CI: −7.05–18.94); this excludes the largest increase ratio in the sixth wave (when the Omicron variant appeared). The average of the case increase ratio for the subsequent epidemic waves after the mass vaccination implementation period was 1.86 (95%CI: 1.04–2.68) (Figure 1B).

From mid-2022 onwards, the seasonal pattern of the epidemic peak was composed of two large epidemic waves each year: the first wave starting in spring (May) and peaking in summer, and the second wave starting in autumn (October to November) and peaking in winter (Figure 1A). The incidence rate of COVID-19 rose sharply to 10.0% in the two epidemic waves in 2022, around one year after vaccination began in Japan. The highest incidence rate (21.2%) was observed in the next epidemic wave in the summer of 2023 and a similar incidence rate (~13%) was observed in the following two seasons.

In 2022, after the seventh epidemic wave, there was no interval before the next epidemic wave; after the weekly incidence rate decreased to 0.2%, the eighth epidemic wave started immediately, and from 2023, the incidence rate remained above 0.2% throughout the year.

The third dose of vaccination reached 67.1% coverage of the whole population, and the fourth dose reached 47.4% coverage by the end of 2022. The fifth, sixth, and seventh doses reached 30.9%, 20.3%, and 14.0%, respectively, between 2023 and early 2024 (Figure 1A). In spite of the decrease in vaccination coverage, the incidence rate of COVID-19 decreased to 7.1% from the end of 2024 and the beginning of 2025 (Figure 1A).

**Figure 3:**
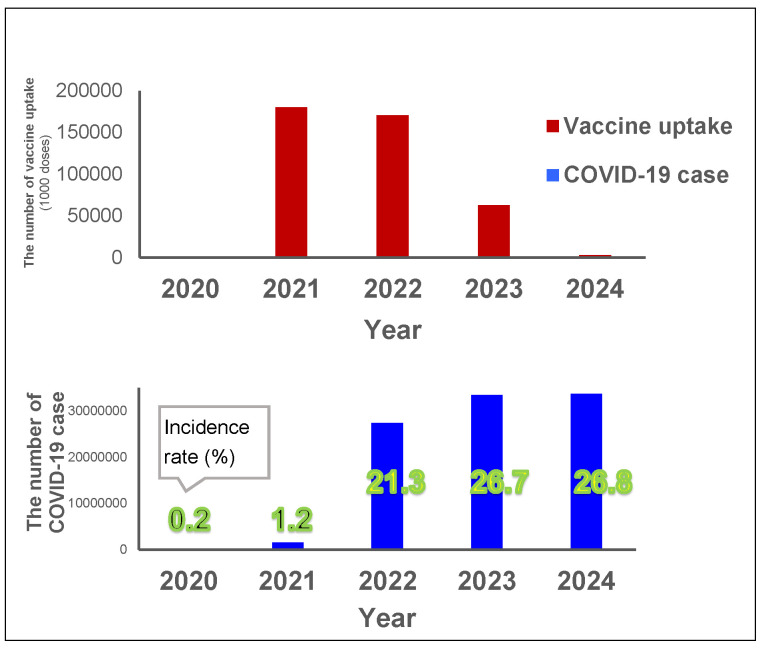
Yearly vaccine uptake and COVID-19 cases. Red bars show the yearly vaccine uptake (unit: 1000 doses) and the blue bars show the yearly number of incidences (and the incidence rate for each year is indicated in green numbers) between 2020 and 2024 (Vaccine data for 2024 was only available until March).

The total vaccination uptake was 0.18 billion doses for first and second doses in 2021, 0.17 billion doses for further doses in 2022, and then decreased to 0.06 billion doses in 2023 (Figure 3). The vaccination in 2021 coincided with a large increase in the incidence rate in 2022, from 1% in 2021 up to 22% in 2022, with an additional 5% increase in incidence rate in 2023 (up to 27%) (Figure 3). The incidence rate in 2024 remained the same (27%) as in 2023.

**Figure 4:**
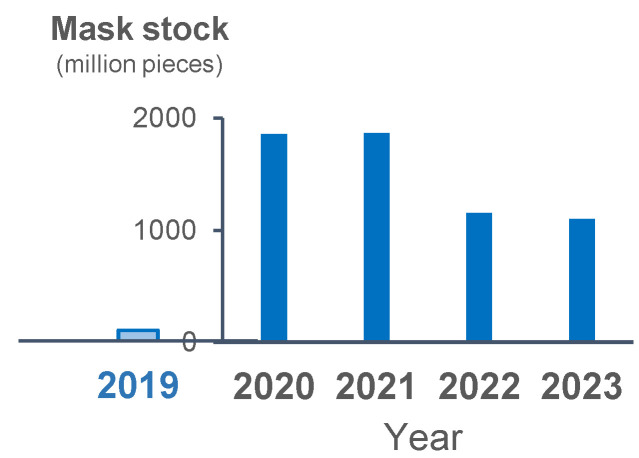
Yearly stock of masks in 2019 and the following 4 years. Blue bars show the yearly stocked number of masks.

The stock of masks increased in 2020 as compared with 2019. The stock of masks was the same between 2020 and 2021, and it slightly decreased but still remained relatively high in 2022 and 2023 (Figure 4). The incidence rate of each epidemic wave decreased from the beginning of 2023, despite the assumed decrease in mask use as suggested by the stock level.

## 4. Discussion

From 2022, there were two epidemic peaks each year, in summer and winter, irrespective of the extent of vaccine coverage or mask use. The weekly case increase ratio in the number of cases started to increase in the winter following the implementation of the mass vaccination program. The fastest increase occurred at the beginning of 2022 with the appearance of the Omicron variant. The case increase ratio decreased relative to this initial surge thereafter, but still remained positive, and the incidence rate increased sharply, with the largest increase occurring in the summer of 2023, suggesting continuous transmission of infection even with a relatively lower case increase ratio. In other words, after the initial vaccination rollout, the incidence rate and the case increase ratio suddenly increased concomitantly, and even after the case increase ratio became lower than the initial period of the pandemic, the incidence rate of infection remained relatively high. Thereafter, the incidence rate decreased even with shrinking vaccination coverage. This sudden increase in the incidence rate despite vaccination with the arrival of the Omicron variant concurs with the evidence suggesting that it is an immune-escape variant [3]. Although it is uncertain the extent to which efficacy was impacted, our results show that the vaccination coverage did not decrease the incidence rate of infection, nor decrease the weekly case increase ratio. Moreover, vaccination roll-out was associated with an increase in the case increase ratio even before the highly transmissible Omicron variant prevailed, suggesting further analysis is needed to examine vaccine efficacy against other variants. There was a decrease in the incidence rate as well as the case increase ratio of the epidemic during the period of lower vaccination coverage, which also coincided with a lower mask stock. This may reflect the acquisition of herd immunity in the population. Following the period when the minimum weekly incidence rate dropped to 0.1–0.2%, the epidemic wave started each time when temperature changes occurred in both the hot and cold seasons.

The occurrence of a winter peak is in line with seasonal patterns of respiratory infection [16]. Our recent study, which compared several influenza epidemics and COVID-19 epidemics, showed that the growth of these respiratory epidemics was similar and that the rate of spread seemed to largely reflect climatic conditions; in winter, the incidence rate exponentially increased irrespective of mask wearing [11]. The occurrence of summer peaks also requires alternative explanations. Such peaks may reflect the acquisition of only short-term immunity, whether induced by the vaccine or naturally acquired, and/or changes in behavior. The fact that these summer peaks occurred irrespective of vaccine coverage calls into question its efficacy. In our previous study regarding influenza, we found that the use of the influenza seroprevalence rates did not predict the decline in incidence rates, potentially suggesting poor immune protection afforded by the antibodies [10]. Although the vaccine efficacy for SARS-CoV-2 has been measured by checking the antibody levels after vaccination [17,18], this alone does not seem to reflect the actual degree of immunity to infection by the virus. Our analysis is clearly limited by correlating patterns of vaccination coverage, mask use, and incidence rates over time without any information on confounding factors such as social activity, testing rates, and actual mask use. However, the in vitro demonstration that Omicron is an immune escape variant concurs with our findings that suggest ineffective vaccine efficacy [3]. With regard to mask use, it is possible that actual mask use in the hot summer months may have decreased, and/or the masks themselves may have become less efficient. However, considering the concern that microplastics in masks have detrimental effects on human health, more detailed studies on the efficacy and durability of different types of masks, as well as their acceptability, need to be carried out. This should then be weighed up against other non-pharmaceutical interventions (e.g., hand-washing, self-imposed isolation when ill) as measures for reducing transmission of respiratory infections prior to the next pandemic [19]. This information would enable a rational allocation of funds to personal protective equipment as opposed to collective measures, including resources for improving response capacity to control outbreaks.

## Data Availability

Data are available in a public, open-access repository, Infectious Diseases Weekly Report (IDWR) https://www.niid.go.jp/niid/en/idwr-e.html (accessed on 20 April 2025).
